# A central role for G9a and EZH2 in the epigenetic silencing of cyclooxygenase-2 in idiopathic pulmonary fibrosis

**DOI:** 10.1096/fj.13-241760

**Published:** 2014-07

**Authors:** William R. Coward, Carol A. Feghali-Bostwick, Gisli Jenkins, Alan J. Knox, Linhua Pang

**Affiliations:** * Division of Respiratory Medicine and; † Nottingham Respiratory Biomedical Research Unit, University of Nottingham, City Hospital, Nottingham, UK; and; ‡ Division of Pulmonary, Allergy, and Critical Care Medicine, University of Pittsburgh School of Medicine, Pittsburgh, Pennsylvania, USA

**Keywords:** histone hypermethylation, DNA methylation, histone deacetylation, antifibrotic gene, lung fibroblasts

## Abstract

Selective silencing of the cyclooxygenase-2 (COX-2) gene with the loss of the antifibrotic mediator prostaglandin E_2_ contributes to the fibrotic process in idiopathic pulmonary fibrosis (IPF). This study explored the role of G9a- and enhancer of zeste homolog 2 (EZH2)-mediated methylation of histone H3 lysine 9 (H3K9me3) and histone H3 lysine 27 (H3K27me3) in COX-2 silencing in IPF. Chromatin immunoprecipitation (ChIP) and re-ChIP assays demonstrated marked increases in H3K9me3, H3K27me3, and DNA methylation, together with their respective modifying enzymes G9a, EZH2, and DNA methyltransferases (Dnmts) and respective binding proteins heterochromatin protein 1 (HP1), polycomb protein complex 1 (PRC1) and methyl CpG binding protein 2 (MeCP2), at the COX-2 promoter in lung fibroblasts from patients with IPF (F-IPFs) compared with fibroblasts from nonfibrotic lungs. HP1, EZH2, and MeCP2 in turn were associated with additional repressive chromatin modifiers in F-IPFs. G9a and EZH2 inhibitors and small interfering RNAs and the Dnmt1 inhibitor markedly reduced H3K9me3 (49−79%), H3K27me3 (44−81%), and DNA methylation (61−97%) at the COX-2 promoter. These reductions were correlated with increased histone H3 and H4 acetylation, resulting in COX-2 mRNA and protein reexpression in F-IPFs. Our results support a central role for G9a- and EZH2-mediated histone hypermethylation and a model of bidirectional, mutually reinforcing, and interdependent crosstalk between histone hypermethylation and DNA methylation in COX-2 epigenetic silencing in IPF.—Coward, W. R., Feghali-Bostwick, C. A., Jenkins, G., Knox, A. J., Pang, L. A central role for G9a and EZH2 in the epigenetic silencing of cyclooxygenase-2 in idiopathic pulmonary fibrosis.

Histones in the chromatin undergo an array of post-translational modifications to regulate gene transcription. Acetylation on lysines by histone acetyltransferases (HATs) and deacetylation by histone deacetylases (HDACs) are associated with transcriptional activation and repression, respectively. The most well-characterized histone methylations are the di- and trimethylation of histone H3 lysine 9 (H3K9me2/3) and histone H3 lysine 27 trimethylation (H3K27me3), which repress gene expression, and histone H3 lysine 4 trimethylation (H3K4me3), which is associated with gene activation (1). H3K9me2/3 is catalyzed by H3K9 specific histone methyltransferases (HMTs), including G9a and SUV39H (2). Methylated H3K9 then serves as a docking site for chromatin modifier proteins that mediate downstream effects, such as heterochromatin protein 1 (HP1), which, in turn, recruits additional HMTs, DNA methyltransferases (Dnmts) and HDAC-containing complexes, such as nuclear receptor corepressor (NCoR), co-RE1-silencing transcription factor (CoREST), and mammalian SIN3 homolog A (mSin3a) to reinforce gene silencing (2). The polycomb group (PcG) proteins are an epigenetic system essentially involved in heritable repression of gene transcription, but the molecular underpinnings are not entirely clear, and several different PcG-mediated mechanisms have been proposed. The enhancer of zeste homolog 2 (EZH2) is the catalytic subunit of the polycomb repressive complex 2 (PRC2), which also includes the suppressor of zeste 12 protein, embryonic ectoderm development (EED) protein, and EED-associated HDAC1 and HDAC2 (3). EZH2 acts as a lysine-specific HMT that mediates H3K27me3; this modification, in turn, provides a specific binding site for another PRC, PRC1, to silence the expression of PRC2 target genes by blocking the recruitment of transcriptional activation factors and preventing initiation of transcription (4, 5). A large percentage of PRC2 target genes contain CpG islands and therefore are likely to undergo DNA methylation as a means to ensure a stable repressive state. There are reports indicating that EZH2 directly interacts with Dnmts and is necessary for *de novo* DNA methylation for PRC2 target gene promoters (6, 7).

DNA methylation by Dnmts at 5-cytosine at CpG islands of gene promoters is the most common epigenetic modification associated with transcriptional silencing. Dnmt3a/b are considered to be the *de novo* Dnmts, whereas Dnmt1 is the primary Dnmt for the maintenance; however, the functions of these Dnmts overlap extensively (8). Direct inhibition of transcription may be through blocking the binding of transcription factors to promoters containing methylated CpG sites (9), whereas indirect repression may involve proteins such as methyl CpG binding protein 2 (MeCP2) that specifically bind to methylated DNA *via* a methyl CpG-binding domain (MBD; ref. 10). Recent studies suggested that intimate communication and mutual dependence exist between DNA methylation and histone modifications in the process of gene silencing. For instance, MBD proteins can bind to methylated DNA and recruit and interact with HDACs and HMTs, thereby linking DNA methylation to histone modifications to reinforce epigenetic silencing (10, 11). However, it is likely that neither of the repressive epigenetic mechanisms can be universally applied to the silencing of specific genes, because this may be dependent on cell type and physiological or pathophysiological context.

Idiopathic pulmonary fibrosis (IPF) is a deadly respiratory disease of unknown etiology with a median survival of 3–4 yr and no effective therapy (12). IPF is characterized by the accumulation and activation of lung fibroblasts and subsequent excessive collagen deposition, leading to distortion of the alveolar architecture, progressive loss of lung function, and ultimately death. Prostaglandin E_2_ (PGE_2_) is a major prostanoid in lung structural cells, including fibroblasts, and is derived mainly from the inducible cyclooxygenase-2 (COX-2; ref. 13, 14). There is compelling evidence that PGE_2_ is a key antifibrotic mediator in the lung because of its inhibition of fibroblast activation and collagen deposition (15). Animal model studies have shown that PGE_2_ and its analog have protective effects against bleomycin-induced pulmonary fibrosis (16, 17); in contrast, a lack of COX-2 and COX-2-derived PGE_2_ promotes fibrosis (18, 19). Moreover, COX-2 expression and PGE_2_ production are markedly reduced in fibroblasts from IPF lung (F-IPFs; ref. 20) and PGE_2_ in bronchoalveolar lavage fluid (21) and COX-2 expression in lung biopsy specimens (22) are also reduced in patients with IPF. These findings suggest that the COX-2/PGE_2_ antifibrotic mechanism is lost in IPF, which in turn promotes fibrosis and contributes to IPF pathogenesis.

We have demonstrated previously that COX-2 gene transcription was defective in F-IPFs compared with that in fibroblasts from nonfibrotic lung (F-NLs) due to deficient histone acetylation as a result of decreased recruitment of HATs and increased recruitment of the HDAC-containing transcriptional corepressor complexes to the COX-2 promoter (13). However, whether histone methylation and DNA methylation affect COX-2 repression in IPF is not known. In this study, we explored the role of G9a- and EZH2-mediated histone methylation and DNA methylation and the interactions between histone modifications and DNA methylation in COX-2 epigenetic silencing in F-IPFs. We report here that G9a-mediated H3K9 methylation and EZH2-mediated H3K27 methylation were markedly increased at the COX-2 promoter in F-IPFs in an interdependent manner and were tightly associated with DNA methylation and histone hypoacetylation, thereby resulting in the epigenetic silencing of the COX-2 gene in F-IPFs. Disruption of G9a- and EZH2-mediated histone methylation by epigenetic inhibitors and G9a and EZH2 small interfering RNAs (siRNAs) reversed repressive epigenetic modifications and restored COX-2 expression and PGE_2_ production. These findings demonstrate a novel and central role for G9a- and EZH2-mediated histone methylation in COX-2 epigenetic silencing in IPF and provide a functional connection between histone methylation and DNA methylation. The intrinsic reversibility and mutual dependence of these epigenetic changes may prove to be beneficial in the reactivation of the antifibrotic COX-2 gene in IPF.

## MATERIALS AND METHODS

### Fibroblast cell culture

F-IPFs and F-NLs from the explanted lungs of patients with IPF who underwent lung transplantation at the University of Pittsburgh Medical Center (Pittsburgh, PA, USA) and from normal lung tissues obtained from organ donors under a protocol approved by the University of Pittsburgh Institutional Review Board were cultured as described previously (23). The 6 F-IPF donors were all male smokers with an average age of 62.95 yr (range, 40.7–74.1 yr) and an average smoking history of 24.17 pack-yr (range, 4–70 pack-yr). The 6 F-NL donors included 3 men, 1 woman, and 2 with undisclosed gender. The age of 1 donor was undisclosed and the average age of the other 5 donors was 42.2 yr (range, 22–63 yr). One donor was a nonsmoker, 1 had a 13-pack-yr smoking history, and the smoking histories of the other 4 donors were undisclosed. F-IPFs and F-NLs from 6 donors each were used at passages 5 and 6, respectively, to ensure purity and maintain the differences present *in vivo*. The cells were growth arrested in serum-free medium for 24 h before stimulation with recombinant human interleukin-1β (IL-1β; 1 ng/ml; R&D Systems, Abingdon, UK) in serum-free medium. At the indicated time points, the cells were harvested for subsequent analysis, and their responses were compared.

### Chromatin immunoprecipitation (ChIP) and re-ChIP assays

ChIP assays were performed using reagents and protocols from a ChIP-IT Express Kit (Active Motif, La Hulpe, Belgium) as described previously (13). Antibodies against H3K4me3, H3K9me2, H3K9me3, H3K27me3, EZH2, acetylated histone H3 and H4, total histone H3 and H4 (EMD Millipore Corp., Billerica, MA, USA), G9a, SUV39H1, HP1 (recognizes HP1α, β, and γ), PRC1, EED, Dnmt1, Dnmt3a, MeCP2 CoREST, NCoR, mSin3a (Santa Cruz Biotechnology, Dallas, TX, USA), and respective control antibodies were used for immunoprecipitation. Purified DNA from the immunoprecipitated antibody-protein-chromatin complexes was subject to real-time PCR amplification with primers designed specifically for the COX-2 promoter region (−299/+6) as described previously (13). The amounts of COX-2 promoter DNA that were present in the bound (immunoprecipitated) fractions were calculated relative to the input control by using the 2^−ΔΔ*C*_*T*_^ method, where ΔΔ*C_T_* is the difference between the threshold cycle (*C_T_*) for the bound fraction and the *C_T_* for the input fraction. The association of acetylated and methylated histones H3 and H4 with the COX-2 promoter DNA was further normalized to the association of total histones H3 and H4 with the COX-2 DNA.

Re-ChIP assays were conducted by using a Re-ChIP-IT Express Kit (Active Motif) as described previously (24). After the first immunoprecipitation using antibody against HP-1, EZH2, or MeCP2, the immunoprecipitated chromatin was removed from the magnetic beads in a buffer that prevents the majority of the first antibody from participating in the second immunoprecipitation reaction. The chromatin was desalted, and a second ChIP step was performed using specific antibodies against NCoR, CoREST, mSin3a, Dnmt3a, Dnmt1, G9a, SUV39H1, EZH2, and PRC1. Purified DNA from the sequentially immunoprecipitated antibody-protein-chromatin complexes was analyzed by real-time PCR as described above.

### Methylated DNA immunoprecipitation (MeDIP) assay

MeDIP assays were performed to detect DNA methylation at the CpG islands within the human COX-2 promoter with the use of a ChIP-IT Express Kit. In brief, after treatment, chromatin was extracted from the cells and portioned into aliquots as described previously (13). Aliquots of chromatin were then incubated with no antibody, a nonimmune rabbit IgG antibody (Santa Cruz Biotechnology) as negative controls, or a specific IgG antibody against 5-methylcytosine (Active Motif) overnight at 4°C. The MeDIP reaction was calibrated with a set of 2 DNA standards (897 bp) that are linear double-stranded DNA with the same sequence (Zymo Research, Irvine, CA, USA; see also the manufacturer's website; http://www.zymoresearch.com). The only difference is that each contains either 100% unmodified cytosines or 5-methylcytosines. Since the sequence and extent of cytosine modification are known, these DNA standards were also used to spike the chromatin to demonstrate the specificity of the MeDIP assay. The immunoprecipitated antibody-DNA complexes were then collected, and DNA was extracted, purified, and subjected to real-time PCR amplification of the minimum COX-2 promoter region (positions −299 to+6) with key transcription factor binding sites (13) and several known CpG sites (25) with specifically designed primers (13). DNA methylation in distal regions upstream and downstream of the minimum COX-2 promoter was also analyzed with specifically designed primers: forward 5′-TCAGCCCAACTGCTTATGTG-3′ and reverse 5′-GGGAGTCATCTCGGTGTGAT-3′) for the region from −12,360 to −12,142; forward 5′-CCCAACAAATTTCAGACGCT-3′ and reverse 5′-TACATTTGGGATGCTGGTCA-3′, for the region from −2440 to −2206; forward 5′-AAGTGGGTGCCATACTCAGC-3′ and reverse 5′-GAGAAGGCTTCCCAGCTTTT-3′ for the region from +1727 to +2093; and forward 5′-CTTCCATCTCCAAGACCCAA-3′ and reverse 5′-TCTTCCTGCTAGGCTACCCA-3′, for the region from +21,087 to +21,307. The amount of COX-2 promoter DNA in the immunoprecipitates (IPs) was calculated as described previously (13). The amounts of COX-2 promoter DNA present in both nonantibody and nonimmune rabbit IgG negative control IPs were minimal and markedly smaller than those in the specific-antibody IPs.

### COX-2 mRNA and protein expression

COX-2 mRNA and protein expression levels in resting and cytokine-stimulated cells were examined by real-time PCR and Western blot, respectively, as reported previously (13, 14).

### Epigenetic inhibitor study

To assess the role of G9a HMT, Dnmt1, and HDACs in COX-2 repression in F-IPFs, inhibitors for these enzymes were used. The G9a HMT-specific inhibitor BIX-01294 [2-(hexahydro-4-methyl-1*H*-1,4-diazepin-1-yl)-6,7-dimethoxy-*N*-[1-(phenylmethyl)-4-piperidinyl]-4-quinazolinamine trihydrochloride hydrate]; ref. 26) and the Dnmt1 inhibitor RG108 (27) were purchased from Sigma-Aldrich (Gillingham, UK). The EZH2 inhibitor 3-deazaneplanocin A (DZNep; ref. 28) was purchased from Cayman Chemical (Ann Arbor, MI, USA). F-NLs and F-IPFs were treated without or with BIX-01294 (100 nM), RG108 (5 μM), or DZNep (10 nM) in medium with serum for 2 d before they reached confluence and then were treated without or with the inhibitors in serum-free medium for 1 d before being incubated without or with IL-1β (1 ng/ml) in the presence or absence of the inhibitors for 4 and 24 h. Samples were then collected for epigenetic analyses by ChIP assay and MeDIP and for COX-2 mRNA and protein expression by real-time RT-PCR and Western blotting, respectively.

### siRNA transfection

All transient transfections were conducted by using HiPerFect Transfection Reagent according to the recommended protocol of the manufacturer (Qiagen, Hilden, Germany). A total of 2 × 10^6^ F-IPFs were seeded into each 100-mm culture dish in culture medium containing serum. Then 600 ng of predesigned siRNA directed against human G9a (target sequence: CACCATGAACATCGATCGCAA, catalog no. SI00091203, Qiagen), EZH2 (target sequence: CAGACGAGCTGATGAAGTAAA, catalog no. SI00063966, Qiagen), or ALLStars negative control siRNA (with no homology to any known mammalian gene, catalog no. 1027280; Qiagen) was diluted in 1.6 ml of culture medium, 48 μl of HiPerFect Transfection Reagent was added to the diluted siRNA, and the preparation was mixed and incubated at room temperature for 10 min. The transfection mixture was added to cells in a 100-mm culture dish in a total volume of 4 ml, and the cells were incubated for 3 h, after which the culture medium was made up to 7 ml with a final siRNA concentration of 5 nM, and cells were incubated for a further 48 h. Cells were then serum starved for 24 h before being treated without or with IL-1β (1 ng/ml) for 2, 4, and 24 h for analyses of epigenetic modifications, G9a, EZH2, and COX-2 mRNA and protein expression, respectively.

### PGE_2_ assay

The PGE_2_ concentration in the culture medium was measured by a commercially available enzyme-linked immunosorbent assay kit (Cayman Chemical).

### Statistics

The data are presented as means ± sem from experiments with 3−6 separate cell lines performed in duplicate. A nonparametric ANOVA was used to evaluate the statistical significance of the mean values between F-NLs and F-IPFs; this was followed by a Wilcoxon rank sum test for comparisons between F-NLs and F-IPFs at specific time points. A value of *P* < 0.05 was considered statistically significant.

## RESULTS

### Histone H3 is repressively methylated at the COX-2 promoter in F-IPFs due to increased G9a and EZH2 recruitment

**Figure 1.**
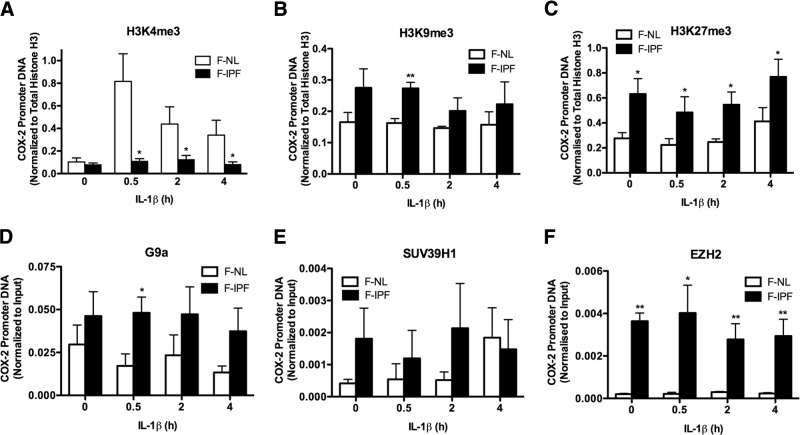
Histone H3 is repressively hypermethylated and HMT recruitment is increased at the COX-2 promoter in F-IPFs. Confluent and serum-starved F-NLs and F-IPFs were incubated with IL-1β (1 ng/ml) for the times indicated. The protein-DNA complexes were cross-linked by formaldehyde treatment, and chromatin pellets were extracted and sonicated. H3K4me3 (*A*), H3K9me3 (*B*), H3K27me3 (*C*), G9a (*D*), SUV39H1 (*E*), EZH2 (*F*), and total histone H3 (*A–C*) were immunoprecipitated with specific antibodies. The associated COX-2 promoter DNA was amplified by real-time PCR, and the amount was calculated and normalized to total histone H3 (*A–C*) or to input control (*D–F*). Data are expressed as means ± sem from experiments with 6 separate F-NL and F-IPF cell lines performed in duplicate. *P < 0.05, **P < 0.005 *vs.* corresponding F-NLs.

We have previously shown that both histones H3 and H4 are insufficiently acetylated at the COX-2 promoter in F-IPFs compared with those in F-NLs (13). Because H3K4me3 and H3K9me3/H3K27me3 are typical epigenetic marks linked to active and repressive chromatin, respectively, we analyzed these marks at the COX-2 promoter by ChIP assay. We found that under unstimulated conditions, H3K4me3 at the COX-2 promoter was slightly lower in F-IPFs than in F-NLs, whereas it was the opposite for H3K9me3 and H3K27me3. Treatment with IL-1β resulted in a marked increase in H3K4me3 in F-NLs (*P*<0.01 at 1 h after stimulation compared with unstimulated) but not in F-IPFs, and the level of H3K4me3 at the COX-2 promoter was significantly lower in F-IPFs than in F-NLs (**Fig. 1*A***). In contrast, under both unstimulated and cytokine-stimulated conditions, H3K9me3 and H3K27me3 at the COX-2 promoter were elevated in F-IPFs compared with those in F-NLs (Fig. 1*B*, *C*). In addition, the association of the 2 major HMTs G9a and EZH2, responsible for H3K9me3 and H3K27me3, respectively (Fig. 1*D*, *F*), but not the HMT SUV39H1, also responsible for H3K9me3 (Fig. 1*E*), with the COX-2 promoter was also consistently increased in F-IPFs compared with that in F-NLs with or without cytokine treatment. The data thus suggest that histone H3K9 and H3K27 are hypermethylated at the COX-2 promoter in F-IPFs as a result of increased recruitment of G9a and EZH2, respectively.

### Recruitment of repressive chromatin modifiers to methylated H3K9 and H3K27 is increased at the COX-2 promoter in F-IPFs

**Figure 2.**
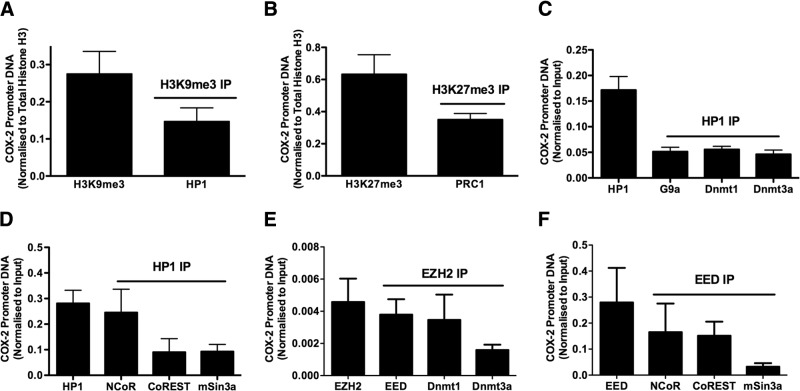
HP1, PRC1, and repressive epigenetic enzymes are associated with the COX-2 promoter in F-IPFs. Confluent F-IPFs were serum starved for 24 h. The protein-DNA complexes were cross-linked by formaldehyde treatment, and chromatin pellets were extracted and sonicated. H3K9me3 (*A*), H3K27me3 (*B*), HP1 (*C* and *D*), EZH2 (*E*), and EED (*F*) were immunoprecipitated with specific antibodies first, and then the IPs were immunoprecipitated again with antibodies against HP1 (*A*), PRC1 (*B*), G9a (*C*), Dnmt1, Dnmt3a (*C* and *E*), EED (*E*), and NCoR, CoREST, and mSin3a (*D*, *F*). The associated COX-2 promoter DNA was amplified by real-time PCR, and the amount was calculated and normalized to input control. Data are expressed as means ± sem from experiments with 6 separate F-IPF cell lines performed in duplicate.

Because methylated histone H3K9 and H3K27 serve as a binding platform for HP1 and PRC1, respectively, we anticipated that the respective association of HP1 and PRC1 with H3K9me3 and H3K27me3 at the COX-2 promoter could be increased in F-IPFs. Using a Re-ChIP assay, we indeed detected HP1 and PRC1 in the IPs of H3K9me3 and H3K27me3, respectively, at the COX-2 promoter under unstimulated conditions (**Fig. 2*A*, *B***). Because HP1 may act as an adapter in mediating histone deacetylation and DNA methylation through its recruitment of the respective epigenetic enzymes, we went on to explore the association of HP1 with G9a, Dnmts, and transcriptional repressors NCoR, CoREST, and mSin3a by a Re-ChIP assay. As shown in Fig. 2*C*, *D*, enrichment of the COX-2 promoter DNA was observed in both primary and secondary IPs in F-IPFs, demonstrating a physical association of HP1 with G9a, Dnmt1, Dnmt3a, NCoR, CoREST, and mSin3a at the COX-2 promoter. EZH2 is the catalytic subunit of PRC2, which also includes EED (3), and has been shown to interact with Dnmts to cause DNA methylation for some gene promoters (6, 7). We found that EZH2 was associated with EED, Dnmt1, and Dnmt3a (Fig. 2*E*) and that EED was associated with NCoR, CoREST, and mSin3a at the COX-2 promoter in F-IPFs (Fig. 2*F*). These observations suggest that G9a-mediated H3K9me3 and EZH2-mediated H3K27me3 are associated with additional chromatin modifiers to the COX-2 promoter through HP1 and EZH2/EED, respectively, which may result in DNA methylation and histone deacetylation and reinforcement of COX-2 epigenetic silencing in F-IPFs.

### COX-2 promoter DNA is methylated in F-IPFs

**Figure 3.**
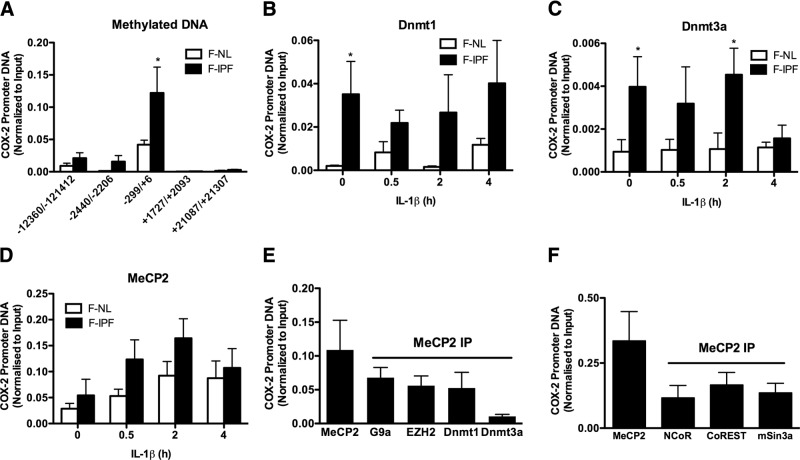
COX-2 promoter DNA methylation and Dnmt association with the COX-2 promoter are increased in F-IPFs. *A*) Confluent F-NLs and F-IPFs were serum starved for 24 h and then lysed. DNA was extracted. Methylated DNA was immunoprecipitated with an antibody against 5-methylcytosine. The associated DNA was amplified by real-time PCR using specific primers for different regions of the COX-2 promoter and its upstream and downstream regions. *B–D*) Confluent and serum-starved F-NLs and F-IPFs were incubated with IL-1β (1 ng/ml) for the times indicated. The protein-DNA complexes were cross-linked by formaldehyde treatment, and chromatin pellets were extracted and sonicated. Dnmt1 (*B*), Dnmt3a (*C*), and MeCP2 (*D*) were immunoprecipitated with specific antibodies. The associated COX-2 promoter DNA was amplified by real-time PCR, and the amount was calculated and normalized to input control. *E*, *F*) Confluent F-IPFs were serum starved for 24 h. The protein-DNA complexes were cross-linked by formaldehyde treatment, and chromatin pellets were extracted and sonicated. MeCP2 was immunoprecipitated with specific antibody first, and then the IPs were immunoprecipitated again with antibodies against G9a, EZH2, Dnmt1, and Dnmt3a (*E*) and NCoR, CoREST, and mSin3a (*F*). The associated COX-2 promoter DNA was amplified by real-time PCR, and the amount was calculated and normalized to input control. Data are expressed as means ± sem from experiments with 6 separate F-NL and/or F-IPF cell lines performed in duplicate. *P < 0.05 *vs.* corresponding F-NLs.

Because the COX-2 promoter is known to contain CpG islands, and COX-2 repression is commonly associated with DNA methylation, we anticipated that the COX-2 promoter DNA could be methylated, thereby contributing to the COX-2 gene repression in F-IPFs. By applying the MeDIP assay with an antibody against 5-methylcytosine and amplifying the immunoprecipitated COX-2 promoter DNA by real-time PCR using specific primers for different regions of the COX-2 promoter, we revealed that the minimum COX-2 promoter region (−299/+6) was significantly more methylated in F-IPFs than in F-NLs under unstimulated conditions (**Fig. 3*A***). The 2 regions upstream (−2440/−2206 and −12,360/−12,142) of the minimum COX-2 promoter were also more methylated in F-IPFs than in F-NLs but to a much lesser extent than that for the minimum COX-2 promoter, whereas no meaningful methylation was observed in the 2 regions downstream (+1727/+2093 and+21,087/+21,307) of the minimum COX-2 promoter (Fig. 3*A*). Because DNA methylation is catalyzed by Dnmts, we next assessed whether the recruitment of Dnmts to the COX-2 promoter was increased in F-IPFs. By using a ChIP assay, we found that the association of Dnmt1 and Dnmt3a with the COX-2 promoter was significantly increased in F-IPFs compared with that in F-NLs under unstimulated conditions and marked, but not statistically significant, increases were maintained after cytokine stimulation (Fig. 3*B*, *C*). However, no difference was observed between F-IPFs and F-NLs in the global protein expression of Dnmt1 and Dnmt3a as analyzed by Western blotting (data not shown). These findings suggest that the COX-2 promoter DNA is methylated in F-IPFs as a result of increased recruitment of Dnmts to the COX-2 promoter rather than increased expression of Dnmt proteins. Because methylated DNA serves as a binding platform for MeCP2, we anticipated that the MeCP2 association with the COX-2 promoter could be increased in F-IPFs. ChIP analysis indeed revealed that there was a trend for increased association of MeCP2 with the COX-2 promoter in F-IPFs compared with that in F-NLs with or without cytokine treatment, although the differences were not statistically significant (Fig. 3*D*). Because MeCP2 can mediate gene silencing through recruitment of chromatin modifiers, we went on to explore the association of MeCP2 with HMTs, Dnmts, and transcriptional repressors by a Re-ChIP assay. As shown in Fig. 3*E*, *F*, enrichment of the COX-2 promoter DNA was observed in both primary and secondary IPs in F-IPFs, demonstrating physical association of MeCP2 with G9a, EZH2, Dnmt1, Dnmt3a, NCoR, CoREST, and mSin3a at the COX-2 promoter and suggesting that DNA methylation, like histone methylation, could also recruit additional epigenetic enzymes to the COX-2 promoter through MeCP2 and lead to reinforced COX-2 epigenetic silencing in F-IPFs.

### Epigenetic inhibitors reduce repressive histone modifications at the COX-2 promoter in F-IPFs

**Figure 4.**
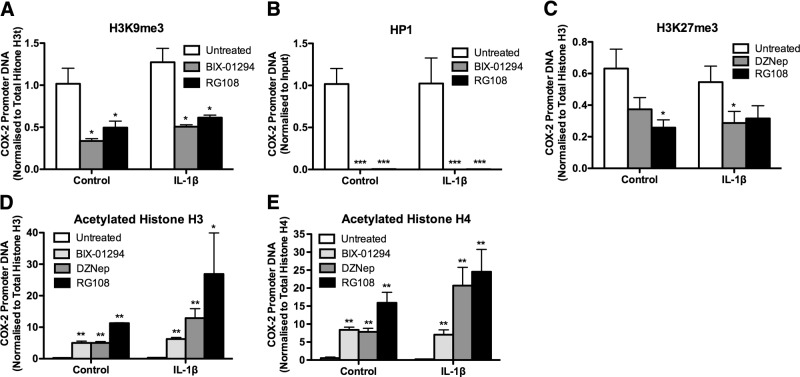
Epigenetic inhibitors of G9a, EZH2, and Dnmt1 reduce H3K9me3 and H3K27me3 and increase histone H3 and H4 acetylation at the COX-2 promoter in F-IPFs. F-IPFs were incubated without or with BIX-01294 (100 nM), RG109 (5 μM), or DZNep (10 nM) in medium with serum for 2 d before they reached confluence and then were treated without or with the inhibitors in serum-free medium for 1 d before being incubated without or with IL-1β (1 ng/ml) in the presence or absence of the inhibitors for a further 4 h. The protein-DNA complexes were then cross-linked by formaldehyde treatment, and chromatin pellets were extracted and sonicated. H3K9me3 (*A*), HP1 (*B*), H3K27me3 (*C*), and acetylated histone H3 (*D*) and H4 (*E*) were immunoprecipitated with specific antibodies. The associated COX-2 promoter DNA was amplified by real-time PCR, and the amount was calculated and normalized to total histone H3 (*A*, *C*, *D*), total histone H4 (*E*), or input control (*B*). Data are expressed as means ± sem from experiments with 6 separate F-IPF cell lines performed in duplicate. *P < 0.05, **P < 0.005, ***P < 0.001 *vs.* corresponding untreated cells.

We have previously shown that HDAC inhibitors increase histone acetylation at the COX-2 promoter site and restore COX-2 expression in F-IPFs (13). To determine whether HMT and Dnmt inhibitors could modulate histone modifications at the COX-2 promoter site, we examined the effect of the G9a inhibitor BIX-01294, the EZH2 inhibitor DZNep, and the Dnmt1 inhibitor RG108 in F-IPFs. Treatment with BIX-01294, either alone or with IL-1β, reduced H3K9me3 at the COX-2 promoter by 65 and 57%, respectively, compared with that in untreated cells, whereas treatment with RG108 reduced H3K9me3 by 49%, either alone or with IL-1β (**Fig. 4*A***); this result was accompanied by reduced HP1 association with the COX-2 promoter (Fig. 4*B*). Similarly, treatment of F-IPFs with DZNep, either alone or with IL-1β, decreased H3K27me3 at the COX-2 promoter by 44 and 49%, respectively, compared with that in untreated cells, whereas treatment with RG108 reduced H3K27me3 by 65 and 46%, respectively (Fig. 4*C*). Treatment of F-IPFs with the inhibitors also resulted in an increase in histone H3 and H4 acetylation without cytokine stimulation compared with that in untreated cells, and a further increase was generally observed after IL-1β stimulation (Fig. 4*D*, *E*). The results show that repressive histone methylation at the COX-2 promoter in F-IPFs can be removed by inhibition of not only the activity of respective HMTs but also the activity of Dnmt1 and that histone acetylation at the COX-2 promoter in F-IPFs can be increased by inhibition of the activity of HMTs and Dnmt1. The data also strongly suggest that G9a and EZH2 play a central role in mediating repressive histone modifications at the COX-2 promoter in F-IPFs and that Dnmt activity is required for this process.

### G9a and EZH2 knockdown alters histone modifications at the COX-2 promoter in F-IPFs

**Figure 5.**
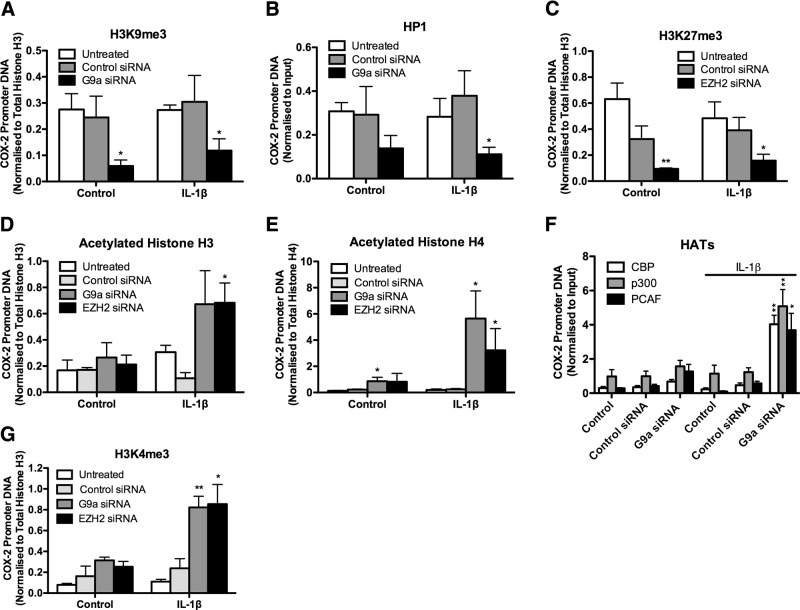
G9a and EZH2 siRNAs reduce H3K9me3 and H3K27me3 and increase histone H3 and H4 acetylation and H3K4me3 at the COX-2 promoter in F-IPFs. F-IPFs were transfected with control siRNA, G9a siRNA, or EZH2 siRNA in medium with serum for 2 d and serum starved for 1 d before being incubated without or with IL-1β (1 ng/ml) in the presence or absence of the siRNAs for a further 4 h. The protein-DNA complexes were then cross-linked by formaldehyde treatment, and chromatin pellets were extracted and sonicated. H3K9me3 (*A*), HP1 (*B*), H3K27me3 (*C*), acetylated histone H3 (*D*) and H4 (*E*), CBP, p300, PCAF (*F*), and H3K4me3 (*G*) were immunoprecipitated with specific antibodies. The associated COX-2 promoter DNA was amplified by real-time PCR, and the amount was calculated and normalized to total histone H3 (*A*, *C*, *D*, *G*), total histone H4 (*E*), or input control (*B*, *F*). Data are expressed as means ± sem from experiments with 6 separate F-IPF cell lines performed in duplicate. *P < 0.05, **P < 0.005 *vs.* corresponding untreated or control cells.

Because pharmacological inhibitors of epigenetic enzymes can have off-target effects, to further validate the role of G9a and EZH2 in mediating COX-2 repression in F-IPFs, we applied siRNA to knock down G9a and EZH2 expression in these cells. Like G9a inhibition by BIX-01294, treatment of F-IPFs with G9a siRNA but not the control siRNA, either alone or with IL-1β, markedly reduced H3K9me3 at the COX-2 promoter by 79 and 60%, respectively, compared with that in untreated cells (**Fig. 5*A***); this result was also accompanied by reduced HP1 association with the COX-2 promoter (Fig. 5*B*). Similarly, like EZH2 inhibition by DZNep, treatment of F-IPFs with EZH2 siRNA, either alone or with IL-1β, resulted in a marked decrease in H3K27me3 (81 and 55%, respectively) at the COX-2 promoter compared with that for untreated cells (Fig. 5*C*). Treatment of F-IPFs with G9a siRNA and EZH2 siRNA also led to a slight increase in histone H3 and H4 acetylation without cytokine stimulation compared with that in untreated cells, and a marked increase was observed after IL-1β stimulation (Fig. 5*D*, *E*); this was accompanied by significant increases in the recruitment of HAT cAMP response element binding protein binding protein (CBP), p300, and p300/CBP-associated factor (PCAF) to the COX-2 promoter (Fig. 5*F*). In addition, G9a siRNA and EZH2 siRNA restored the response of F-IPFs to IL-1β to increase the active histone modification H3K4me3 at the COX-2 promoter (Fig. 5*G*). The data confirm the effect of G9a and EZH2 inhibitors in Fig. 4 and a central role for G9a and EZH2 in mediating repressive histone modifications at the COX-2 promoter in F-IPFs.

### G9a and EZH2 are required for DNA methylation at the COX-2 promoter in F-IPFs

**Figure 6.**
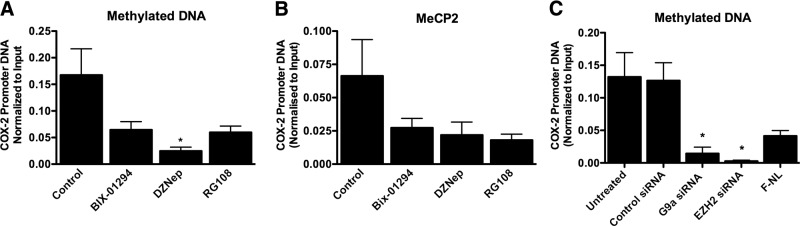
G9a and EZH2 inhibitors and siRNAs reduce COX-2 promoter DNA methylation in F-IPFs. *A*, *B*) F-IPFs were incubated without or with BIX-01294 (100 nM), DZNep (10 nM), or RG109 (5 μM) in medium with serum for 2 d before they reached confluence and then were treated without or with the inhibitors in serum-free medium for 1 d. Cells were then lysed, and DNA was extracted and sheared. *A*) Methylated DNA was immunoprecipitated with an antibody against 5-methylcytosine. The associated DNA was amplified by real-time PCR using specific primers for the COX-2 promoter DNA. *B*) The protein-DNA complexes were cross-linked by formaldehyde treatment and chromatin pellets were extracted and sonicated. MeCP2 was immunoprecipitated with a specific antibody. The associated COX-2 promoter DNA was amplified by real-time PCR, and the amount was calculated and normalized to the input control. *C*) F-IPFs were transfected with control siRNA, G9a siRNA, or EZH2 siRNA in medium with serum for 2 d and serum starved for 1 d. The cells were then lysed, and DNA was extracted and sheared. Methylated DNA was immunoprecipitated with an antibody against 5-methylcytosine. The associated DNA was amplified by real-time PCR using specific primers for the COX-2 promoter DNA with samples from F-NLs as a reference. Data are expressed as means ± sem from experiments with 6 separate F-IPF and F-NL cell lines performed in duplicate. *P < 0.05 *vs.* control cells.

Because Dnmt activity could be required for G9a- and EZH2-mediated histone H3 hypermethylation at the COX-2 promoter in F-IPFs (Fig. 4*A*, *C*), we went on to assess whether G9a and EZH2 could be required for COX-2 promoter DNA methylation. As shown in **Fig. 6*A***, treatment of the cells with either BIX-01294, DZNep, or RG108 reduced DNA methylation by 62, 83, and 64%, respectively, at the minimum COX-2 promoter region (−299/+6), with significant inhibition by DZNep. The inhibitors also similarly reduced the binding of MeCP2 to the COX-2 promoter, although the reduction was not statistically significant (Fig. 6*B*). Furthermore, both G9a and EZH2 siRNAs but not the control siRNA also markedly reduced DNA methylation at the minimum COX-2 promoter by 86 and 97%, respectively, compared with that for untreated F-IPFs, to a level lower than that observed with F-NLs (Fig. 6*C*), thereby confirming the effect of the G9a and EZH2 inhibitors. The combined results of Figs. 4 and 6 thus suggest that G9a and EZH2 are critically involved in COX-2 promoter DNA methylation and that histone methylation and DNA methylation act interdependently to cause COX-2 epigenetic silencing in IPF cells; removal of one of these repressive epigenetic modifications leads to the removal of the other repressive epigenetic modifications and consequently the derepression of the COX-2 gene.

### G9a and EZH2 inhibition and disruption restore COX-2 expression in F-IPFs

**Figure 7.**
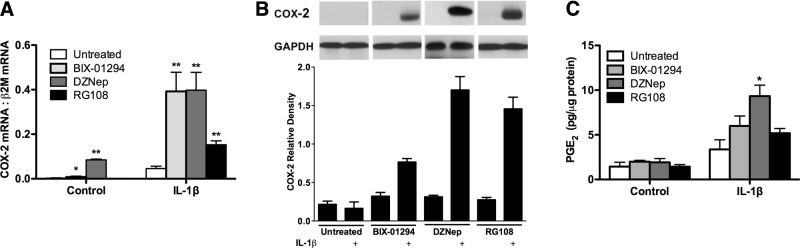
Epigenetic inhibitors of G9a, EZH2, and Dnmt1 restore COX-2 expression and PGE_2_ production in F-IPFs. F-IPFs were incubated without or with BIX-01294 (100 nM), DZNep (10 nM), or RG108 (5 μM) in medium with serum for 2 d before they reached confluence and then were treated without or with the inhibitors in serum-free medium for 1 d before being incubated without or with IL-1β (1 ng/ml) in the presence or absence of the inhibitors for a further 4 h (*A*) or 24 h (*B*, *C*). *A*) Total RNA was isolated, and mRNA levels of COX-2 and the internal control β_2_-microglobulin (β2M) were determined by real-time RT-PCR. The data are calculated as the ratio of COX-2 mRNA and β2M mRNA and are expressed as means ± sem of 6 separate experiments performed in duplicate. *B*) Total cell lysates were collected for Western blotting analysis of COX-2 with GAPDH as the loading control. Data are representative of 3 separate experiments with different F-IPF cell lines. Relative density was calculated by normalizing the density of the COX-2 bands against that of the GAPDH bands from 3 separate experiments. *C*) Culture media were collected for PGE_2_ assay. For panels *A*, *C*, data are expressed as means ± sem from experiments with 6 separate F-IPF cell lines performed in duplicate. *P < 0.05, **P < 0.005 *vs.* corresponding untreated cells.

To investigate whether G9a and EZH2 inhibition could lead to COX-2 gene derepression, F-IPFs were treated with BIX-01294, DZNep, or RG108, either alone or with the potent COX-2 inducer IL-1β. BIX-01294 and DZNep alone, but not RG108, caused a slight but significant increase in COX-2 mRNA expression, as analyzed by real-time RT-PCR, whereas marked increases in COX-2 mRNA were observed when the cells were treated with BIX-01294, DZNep, or RG108 and IL-1β together, and marked increases in COX-2 mRNA (by 8.57-, 7.25-, and 3.34-fold, respectively) were observed compared with that for IL-1β alone (**Fig. 7*A***). Western blotting analysis revealed no COX-2 protein expression when the cells were treated with the inhibitors or with IL-1β alone; however, when the cells were treated with the inhibitors and IL-1β together, marked increases in COX-2 protein expression were observed (Fig. 7*B*). To find out whether the restoration of COX-2 protein expression in F-IPFs was accompanied by PGE_2_ production, PGE_2_ in the medium was also analyzed. As shown in Fig. 7*C*, no increase in PGE_2_ production was observed when the cells were treated with the inhibitors alone, and IL-1β alone stimulated a small increase in PGE_2_ production; however, when the cells were treated with IL-1β and BIX-01294, DZNep, or RG108, further increases by 1.78-, 2.77-, and 1.54-fold, respectively, were observed compared with those for IL-1β alone. The results show that cytokine-induced COX-2 expression in F-IPFs can be restored by inhibition of the activity of either G9a, EZH2, or Dnmt1, leading to the reproduction of PGE_2_.

**Figure 8.**
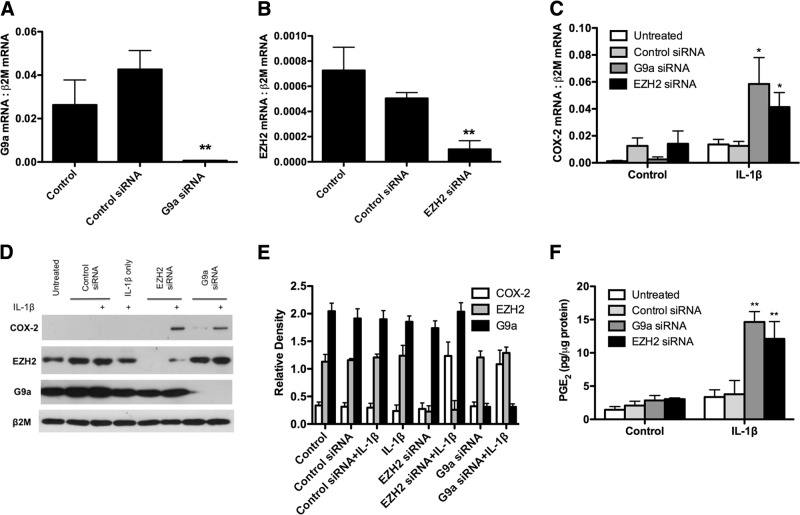
G9a and EZH2 siRNAs restore COX-2 expression and PGE_2_ production in F-IPFs. *A*, *B*) F-IPFs were transfected with control siRNA, G9a siRNA, or EZH2 siRNA in medium with serum for 2 d and serum starved for 1 d. Total RNA was isolated, and mRNA levels of G9a (*A*) and EZH2 (*B*) were determined by real-time RT-PCR. The data are calculated as the ratio of G9a and EZH2 mRNA and the internal control β_2_-microglobulin (β2M) mRNA. *C−F*) F-IPFs transfected with or without siRNAs were serum starved for 1 d before being incubated without or with IL-1β (1 ng/ml) for a further 4 h (*C*) or 24 h (*D−F*). *C*) Total RNA was isolated, and mRNA levels of COX-2 were determined by real-time RT-PCR. The data are calculated as the ratio of COX-2 mRNA and the internal control β2M mRNA. *D*) Total cell lysates were collected for Western blotting analysis of COX-2, G9a, and EZH2 with β2M as the loading control. Data are representative of 3 separate experiments with different F-IPF cell lines. *E*) Relative density of the Western blot was calculated by normalizing the density of the COX-2, EZH2, and G9a bands against that of the β2M bands from 3 separate experiments. *F*) Culture media were collected for PGE_2_ assay. For panels *A−C* and *F*, data are expressed as means ± sem from experiments with 6 separate F-IPF cell lines performed in duplicate. *P < 0.05, **P < 0.005 *vs.* corresponding control or untreated cells.

To explore the effectiveness of G9a and EZH2 siRNA in F-IPFs, G9a and EZH2 mRNA expression was analyzed after siRNA transfection. As shown in **Fig. 8*A*, *B***, G9a and EZH2 siRNAs, but not the control siRNA, reduced their respective mRNAs by 96 and 76% compared with those in the control cells. Knocking down G9a and EZH2 with the siRNAs alone did not increase COX-2 mRNA; however, when the cells were stimulated with IL-1β, marked increases in COX-2 mRNA (by 6.77- and 4.26-fold, respectively) were observed compared with those for IL-1β alone (Fig. 8*C*). Western blotting also revealed that G9a and EZH2 siRNAs almost abolished the expression of their respective proteins, whereas the control siRNA and IL-1β had no effect (Fig. 8*D*). No COX-2 protein was detected in cells treated with G9a and EZH2 siRNAs or IL-1β alone; however, when the cells were stimulated with IL-1β, marked increases in COX-2 protein expression were observed (Fig. 8*D*). The results therefore strongly suggest that G9a and EZH2 play a central role in mediating the epigenetic silencing of COX-2 in IPF.

## DISCUSSION

We have previously demonstrated that histone deacetylation as a result of decreased recruitment of HATs and increased recruitment of HDACs to the COX-2 promoter is involved in COX-2 silencing in F-IPFs (13). Although histone methylation has been shown to act cooperatively with histone acetylation and DNA methylation to determine a heritable transcriptional state, the role of histone methylation in the repression of antifibrotic genes, including COX-2, in IPF has not been explored so far. The current study demonstrated that histone H3 was repressively methylated at the COX-2 promoter as manifested by reduced H3K4me3 and increased H3K9me3 and H3K27me3 in F-IPFs compared with those in F-NLs, which correlated with the recruitment of the H3K9-specific HMT G9a and the H3K27-specific HMT EZH2. Disruption of the function and expression of G9a and EZH2 by their respective inhibitors and siRNAs resulted in marked reductions in H3K9me3 and H3K27me3 and in DNA methylation at the COX-2 promoter and led to the restoration of COX-2 expression and PGE_2_ production. The data strongly suggest that G9a- and EZH2-mediated histone methylation plays a central role and acts interdependently with DNA methylation in the epigenetic silencing of COX-2 in IPF.

It is well established that G9a- and EZH2-mediated histone H3K9 and H3K27 hypermethylation contributes to the epigenetic silencing of tumor suppressor genes. G9a and EZH2 are up-regulated in a number of different cancers (29, 30) and seem to be required for the maintenance of the malignant phenotype. However, G9a expression may not be correlated with global H3K9 methylation (31). We have previously reported that G9a and H3K9me3 are markedly increased at the promoter of another repressed antifibrotic gene, IFN-γ-inducible protein of 10 kDa (IP-10), in F-IPFs, but global G9a expression remains the same between F-IPFs and F-NLs (24). In the current study, we found no differences in the mRNA levels of EZH2 between F-IPFs and F-NLs (data not shown); the increase in H3K9 and H3K27 methylation at the COX-2 promoter in F-IPFs was therefore probably due to gene promoter-specific recruitment of G9a and EZH2.

G9a-mediated H3K9 hypermethylation (both H3K9me2 and H3K9me3) serves as a docking site for the chromatin modifier protein HP1, which in turn recruits additional chromatin modifiers to reinforce gene silencing (2). On the other hand, EZH2-mediated H3K27me3 provides a specific binding site for PRC1 to silence the expression of PRC2 target genes by blocking the recruitment of transcriptional activation factors and preventing initiation of transcription (4, 5). EZH2 also interacts with Dnmts, and another PRC2 protein, EED, interacts with HDAC1 and HDAC2 (3); both are necessary for a stable repressive state for PRC target gene promoters (6, 7). We revealed that HP1 was associated with H3K9me3, G9a, Dnmt1, Dnmt3a, and the transcriptional repressor complexes CoREST and mSin3a (containing HDAC1 and HDAC2) and NCoR (containing HDAC3) at the COX-2 promoter in F-IPFs, suggesting that G9a-mediated H3K9 methylation may lead to DNA methylation and histone deacetylation to reinforce the epigenetic silencing of the COX-2 gene. This observation is supported by our previous findings that interactions between G9a-mediated H3K9 methylation and histone deacetylation are critically involved in the repression of IP-10 in IPF (24) and suggests that similar epigenetic deregulation may account for the silencing of a group of antifibrotic genes in IPF. We also noticed that PRC1 was associated with EZH2-mediated H3K27me3, EZH2 was associated with EED, Dnmt1, and Dnmt3a, and EED was associated with NCoR, CoREST, and mSin3a at the COX-2 promoter in IPF, suggesting that COX-2 silencing in IPF could be mediated by PRC1 through blocking of transcriptional activation factor recruitment and preventing transcription initiation and that EZH2/EED may also lead to DNA methylation and histone deacetylation to reinforce epigenetic silencing of the COX-2 gene. To the best of our knowledge, this is the first report to show PRC-mediated COX-2 epigenetic silencing in any cell system.

To further assess the role of G9a and EZH2 in the epigenetic silencing of the COX-2 gene in F-IPFs, we studied the effects of the G9a-specific inhibitor BIX-01294 and the EZH2 inhibitor DZNep. We found that BIX-01294 significantly reduced not only H3K9me3 and HP1 binding but also DNA methylation and MeCP2 binding and increased histone H3 and H4 acetylation at the COX-2 promoter. Similarly, DZNep reduced not only H3K27me3 but also DNA methylation and MeCP2 binding and increased histone H3 and H4 acetylation at the COX-2 promoter. These results are consistent with previous findings showing that BIX-01294 reduced H3K9 methylation and HP1 recruitment but increased histone H3 and H4 acetylation at the IP-10 promoter in F-IPFs (24). However, epigenetic inhibitors may have off-target effects as demonstrated by a recent study showing inhibition of both EZH2 and the H3K9 HMT SETDB1 by DZNep (28). By using the G9a inhibitor BIX-01294, we observed a total loss of HP1 binding after ∼60% reduction in H3K9me3, whereas G9a siRNA-reduced HP1 binding was proportional to the reduction in H3K9me3. Although the reasons for this discrepancy are not known, it is possible that this may be an off-target effect of BIX-01294. The variable levels of effects of epigenetic inhibitors on COX-2 mRNA/protein expression and PGE_2_ production despite similar effects on methylation status at the COX-2 promoter may also suggest off-target effects of the inhibitors on genes that may have control of COX-2 expression and function. It is therefore necessary to apply siRNA knockdown to validate the findings with epigenetic inhibitors. We revealed in our current study that successful siRNA knockdown of G9a and EZH2 recapitulated the effects of G9a and EZH2 inhibitors. This finding is consistent with recent findings that G9a knockdown reduces H3K9 methylation and the recruitment of HP1, Dnmt1, and HDAC1 to the promoter of the cell adhesion molecule Ep-CAM and restores its expression in lung cancer cells (31) and that G9a knockdown restores E-cadherin expression by suppressing H3K9 methylation and blocking DNA methylation in human breast cancer cells (32). As a result of the removal of epigenetic repression by inhibition and knockdown of G9a and EZH2, active epigenetic modifications such as histone acetylation as a result of increased HAT recruitment and the capability of F-IPFs to express COX-2 and produce the antifibrotic mediator PGE_2_ in response to IL-1β were restored. The data thus strongly suggest that G9a- and EZH2-mediated histone methylation is closely associated with DNA methylation and histone deacetylation and that G9a and EZH2 inhibition and knockdown lead to decreased recruitment of repressive chromatin modifiers to the COX-2 promoter and the switching of local epigenetic status from repression to activation.

It has been demonstrated that DNA methylation-mediated transcriptional silencing is a predominant mechanism for COX-2 silencing in various tumors and that the methylation inhibitor 5-deoxy-2′-azacytidine restores COX-2 gene expression (33, 34). The current study demonstrated that the COX-2 promoter was markedly more methylated in F-IPFs than in F-NLs, which was correlated with increased recruitment of Dnmt1 and Dnmt3a. Because there was no difference in global Dnmt1 and Dnmt3a protein expression between F-NLs and F-IPFs (data not shown), DNA methylation at the COX-2 promoter in F-IPFs was probably due to gene-specific recruitment of Dnmts. Similarly, global DNA methylation does not contribute to the methylation of the promoter of the antifibrotic gene Thy-1 in lung fibroblasts with repressed Thy-1 expression (35). DNA methylation can also lead to histone modifications to establish stable gene silencing through the recruitment of additional chromatin modifiers by proteins with methyl DNA binding activity, such as MeCP2 (10, 11). However, the role of such interactions in COX-2 repression has not been understood. Our current study showed that the increase in DNA methylation was correlated with increased H3K9me3 and H3K27me3 and decreased H3 and H4 acetylation at the COX-2 promoter in F-IPFs, suggesting that DNA methylation and histone modifications act cooperatively in COX-2 repression in IPF. We also presented evidence that there was a trend for increased association of MeCP2 with the COX-2 promoter in F-IPFs compared with that for F-NLs and that MeCP2 was associated with G9a, EZH2, Dnmt1, Dnmt3a, NCoR, CoREST, and mSin3a at the COX-2 promoter, suggesting that DNA methylation/MeCP2-mediated recruitment of chromatin modifiers may reinforce H3K9 and H3K27 methylation, histone deacetylation, DNA methylation, and eventually COX-2 silencing. This result was supported by the findings that the Dnmt1 inhibitor RG108 not only inhibited DNA methylation and MeCP2 binding but also reduced H3K9 and H3K27 methylation and increased histone H3 and H4 acetylation at the COX-2 promoter, eventually resulting in COX-2 derepression in F-IPFs. Although Dnmt1 represents the preferential target of RG108 because the catalytic domains of all 3 Dnmts are highly conserved, they are likely to have similar interactions with RG108 (27). There is also evidence that the functions of these Dnmts overlap extensively (8), making it impractical to knock down individual Dnmts. These observations suggest that the effect of RG108 observed in this study could be attributed to its inhibition of all 3 Dnmts and/or to its inhibition of the Dnmt1 activities on both *de novo* methylation and maintenance of methylation. However, it is also possible that the inhibition of H3K9 and H3K27 methylation by RG108 at the COX-2 promoter could be a direct effect because it has been shown that another Dnmt inhibitor, 5-aza-2′-deoxycytidine could act directly on G9a and EZH2 to reduce H3K9me3 and H3K27me3 at the Bad promoter (36). Nevertheless, the fact that MeCP2 is associated with HMTs and HDACs supports a bidirectional and reinforcing interaction between DNA methylation and histone methylation in the epigenetic silencing of COX-2 in IPF.

It has been increasingly recognized that F-IPFs represent a persistently activated phenotype of lung fibroblasts and play a key role in the development and progression of IPF. Evidence is emerging to support the concept that these cells are epigenetically reprogramed so that a group of antifibrotic genes, including COX-2, Thy-1, and IP-10, are epigenetically silenced in a way similar to the epigenetic silencing of tumor suppressor genes in cancers. Here we provide further evidence that deregulated histone and DNA methylation contributes to the epigenetic silencing of COX-2 in F-IPFs. However, one clear limitation of the current study is that it explored the epigenetic events at the end of the disease process. Although the results are novel and valuable in understanding the epigenetic mechanisms involved in the epigenetic silencing of antifibrotic genes in IPF, little is known about the causes and order of events by which repressive patterns of histone and DNA methylation are established and maintained during the disease development and progression. It is of great importance to identify the critical initiating event that triggers antifibrotic gene silencing to understand the mechanisms underlying persistent fibroblast activation and pulmonary fibrogenesis. It is also plausible to propose that environmental and endogenous factors represent key signals that trigger distinct epigenetic changes and influence the order of epigenetic events, leading to antifibrotic gene silencing during pulmonary fibrogenesis. Preliminary data from our ongoing studies show that chronic treatment of F-NLs with the profibrotic cytokine transforming growth factor-β1 (TGFβ1) markedly reduces IL-1β-induced COX-2 expression, which is accompanied by increased DNA methylation and decreased histone H3 and H4 acetylation at the COX-2 promoter, and that PGE_2_ not only prevents TGFβ1-induced COX-2 repression in F-NLs but also restores COX-2 expression in F-IPFs (unpublished data). These observations provide a hint that repressive epigenetic modifications at the COX-2 promoter could be introduced, at least to some extent, by TGFβ1 treatment and that endogenously produced PGE_2_ after COX-2 reexpression could play a key role in preventing and reversing lung fibroblast activation in IPF. However, further studies are needed to understand the mechanisms and order of epigenetic events leading to the epigenetic silencing of COX-2 and other antifibrotic genes in IPF.

**Figure 9.**
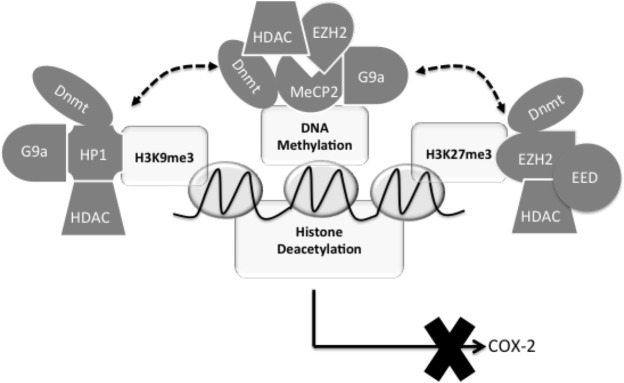
A hypothetical model depicting the central role of G9a- and EZH2-mediated histone methylation and the interdependent and mutually reinforcing crosstalk between histone methylation and DNA methylation in COX-2 epigenetic silencing in IPF. G9a- and EZH2-mediated H3K9me3 and H3K27me3 result in the recruitment of Dnmts and HDAC-containing complexes *via* HP1 and EZH2/EED, respectively, to the COX-2 promoter, which then leads to or reinforces DNA methylation and histone deacetylation. DNA methylation in turn causes the recruitment of G9a, EZH2, and HDAC-containing complexes through MeCP2 to strengthen H3K9me3, H3K27me3, and histone deacetylation, leading to reinforced epigenetic silencing of the COX-2 gene in IPF. Therefore, G9a- and EZH2-mediated H3K9me3 and H3K27me3 interact with DNA methylation in a bidirectional and mutually dependent manner to reinforce COX-2 epigenetic silencing in IPF. Disruption of any of these epigenetic modifications by inhibition or knockdown of G9a, EZH2, or Dnmt leads to the removal of the other repressive epigenetic modifications, resulting in an active chromatin state and reactivation of COX-2 in IPF.

Regardless of the hierarchical order of events, our observations support a central role for G9a- and EZH2-mediated histone hypermethylation and a model of bidirectional, mutually reinforcing, and interdependent crosstalk between histone hypermethylation and DNA methylation in the epigenetic silencing of COX-2 and potentially other antifibrotic genes in IPF (**Fig. 9**). Our data also suggest that the machinery for the expression of COX-2 and probably other antifibrotic genes in IPF remains intact and functional and can mediate reexpression if the repressive epigenetic modifications are removed. Thus, the epigenetic enzymes regulating these epigenetic modifications and their crosstalk in these cells may represent a key therapeutic target to reactivate silenced antifibrotic genes for treating this progressive and fatal disease.
